# Inspiratory muscle resistance combined with strength training: effects on aerobic capacity in artistic swimmers

**DOI:** 10.3389/fspor.2024.1476344

**Published:** 2024-12-04

**Authors:** Yuncai Fan, Yucheng Duan, Zhiqing Gao, Yong Liu

**Affiliations:** Performance Evaluation and Integrated Enhancement Lab, Beijing Research Institute of Sports Science, Beijing, China

**Keywords:** inspiratory muscle resistance training, combined with strength training, aerobic capacity, artistic swimmers, inspiratory muscle resistance combined with strength training

## Abstract

**Objective:**

This study aimed to investigate the effects of combining inspiratory muscle resistance with strength training on lung function and aerobic capacity in artistic swimmers.

**Methods:**

This research constitutes a placebo-controlled randomized trial, involving a five-week walking program conducted twice a week. Fourteen female artistic swimmers were randomly assigned to either the experimental group (TG group, *n* = 7) or the control group (CG group, *n* = 7). Both groups underwent the same strength training program. The TG group performed inspiratory muscle strengthening at 50% of maximum inspiratory pressure (MIP) during strength training, whereas the CG group engaged in strength training with 15% MIP inspiratory muscle training twice a week. Pre- and post-intervention assessments included an incremental stress test, pulmonary function test, inspiratory muscle strength test, and a specialized performance test.

**Results:**

Following the 5-week intervention, within-group comparisons revealed that the inspiratory muscle strength index, vital capacity, and forced vital capacity significantly increased in both the CG and TG groups (*P* < 0.01). The TG group exhibited a significant increase in minute ventilation (*P* < 0.05), while the CG group showed a significant increase in tidal volume (*P* < 0.05). Additionally, the TG group demonstrated a significant improvement in running speed at the ventilation threshold and maximum oxygen uptake (*P* < 0.05), along with a significant increase in tidal volume (*P* < 0.01). Both the CG and TG groups showed significant increases in the scores for the 50-meter diving and 25-meter torpedo (*P* < 0.05). Between-group comparisons showed that the TG group experienced significant improvements in the inspiratory muscle strength index, minute ventilation, running speed corresponding to maximum oxygen uptake, and 25-meter torpedo performance (*P* < 0.05).

**Conclusion:**

The findings suggest that a 5-week program combining inspiratory muscle resistance with strength training can effectively enhance the aerobic exercise capacity of artistic swimmers. Moreover, high-intensity inspiratory muscle resistance combined with strength training can improve the muscle's ability to utilize oxygen during exercise.

**Trial Registration:**

Beijing Research Institute of Sports Science (TKSLL202201); China Clinical Trial Registry, ChiCTR2300072833, 26/06/2023.

## Introduction

Artistic swimming perform strenuous underwater exercise during prolonged breath holds. Approximately 50%–65% of the movements in a artistic swimmer's routine require prolonged breath-holding in the water, coupled with the completion of high-intensity physical leg combinations and treading actions. This necessitates athletes to maintain a high vital capacity and low breathing rate simultaneously, placing heightened demands on inspiratory muscle strength. Studies on the control of ventilation in elite artistic swimmers show that elite artistic swimmers have increased lung volumes, blunted hypoxic ventilatory responses, and a marked apneic bradycardia that may provide physiological characteristics that offer a competitive advantage for championship performance (1, 2). The latest regulations for artistic swimming emphasize the need for standardized choreography and design, alongside quantifying action scores, resulting in an escalating complexity in movement sets. This includes an increase in the number and difficulty of lifts, as well as the complexity and duration of leg combinations, presenting significant challenges to athletes' physical fitness.Studies on the control of ventilation in elite artistic swimmers show that elite artistic swimmers have increased lung volumes, blunted hypoxic ventilatory responses, and a marked apneic bradycardia that may provide physiological characteristics that offer a competitive advantage for championship performance (1, 2). The latest regulations for artistic swimming emphasize the need for standardized choreography and design, alongside quantifying action scores, resulting in an escalating complexity in movement sets. This includes an increase in the number and difficulty of lifts, as well as the complexity and duration of leg combinations, presenting significant challenges to athletes' physical fitness.

Numerous studies have indicated that inspiratory muscle training can enhance ventilation efficiency by augmenting inspiratory explosiveness and tidal volume, aligning with the respiratory requirements of artistic swimming events. In the García I study (3, 4), breath-holding swimming training increased pulmonary diffusion capacity (+9.2%) compared to pre-training, and have greater lung capacity and diffusion (the efficiency of oxygen and carbon dioxide in the alveolar-capillary membrane for gas exchange) than terrestrial athletes. Because of the program's characteristic of repeated apnea in an aquaerous environment, which is equivalent to breath-holding training, and this type of training requires a low respiratory rate and high tide volume,a breathing pattern that involves greater inspiratory muscle strength (5).Inspiratory muscle training(IMT) has been demonstrated to enhance ventilation efficiency by increasing inspiratory explosiveness and tidal volume (6–8), aligning with the specific breathing demands of artistic swimming.This training has been applied to some endurance sports now. In the current study, the training method adopted for inspiratory muscle training is constant load training. Fernández-Lázaro D, study (9, 10) showed that PowerBreath is a useful device to stimulate sport performance and increase pulmonary function. The IMT equipment is designed based on resistance training principles, similar to using dumbbells for arm strength. By gradually increasing inspiratory resistance as strength improves, it is reasonable to assume that this method can effectively enhance IM strength, improve inspiratory muscle explosive force, breath-holding ability, and ultimately, enhance pulmonary function and exercise performance in Artistic swimmers, similar to the effects observed in arm strength training.

The primary objective of this study is to examine the effect of a 5-week combined regimen of inspiratory muscle resistance and strength training on enhancing aerobic capacity in artistic swimmers. It aims to provide methodological support for improving the overall competitive ability of artistic swimmers.

## Method

### Participants

The Beijing artistic swimming team consists of 14 female athletes (mean age 19.9 ± 3.1years) renowned for their collective event championships in national artistic swimming competitions. Prior to the experimental phase, all participants underwent a thorough screening process, ensuring they were non-smokers with no history of disease, metabolic or endocrine disorders, and possessed normal pulmonary function. Detailed information about the experiment's objectives, training procedures, potential physical discomfort, and the voluntary nature of their participation was provided to the subjects. Furthermore, participants were required to provide informed consent and pledge full cooperation throughout the experiment. Using a random number table, the subjects were divided into a control group (CG) and an experimental group (TG), each comprising 7 individuals. This allocation resulted in comparable basic information between the two groups (*P* > 0.05), ensuring data comparability, group-specific details outlined in Table 1. All procedures were approved by Beijing Research Institute of Sports Science (TKSLL202201) and the ethics committee of Chinese Clinical Trail Registry (Approval number: ChiCTR2300072833).

**Table 1:** Basic information of subjects recruited.

Group	Height (cm)	Weight (kg)	Age 9 years)	training experience (years)
CON (*n* = 7)	166.5 ± 3.5	51.7 ± 3.5	20.6 ± 3.1	11.1 ± 3.0
EXP (*n* = 7)	167.3 ± 3.2	50.9 ± 4.5	19.0 ± 2.8	10.9 ± 2.7

### Study design

This research constitutes a placebo-controlled randomized trial, involving a five-week walking program conducted twice a week. The TG group underwent combined inspiratory muscle strength training at an intensity of 50% maximal inspiratory pressure (MIP), while the CG group engaged in the same training at 15% MIP intensity. Both groups followed artistic breathing patterns and coordinated physical activity during training, focusing on utilizing abdominal breathing and ensuring forceful inhalation during exercises in synchronization with strength training movements. Each set comprised 8 movements with 6–8 repetitions per movement, totaling 3 sets with 60-second intervals between each set. Furthermore, participants exhaled during exertion and inhaled during the reset phase of each movement to maintain consistent breathing patterns throughout the training sessions.The subjects' maximum oxygen uptake, resting lung function, inspiratory muscle strength, and swimming performance were evaluated before and after the training phase, See Table 2 for details.

**Table 2:** Specialized strength training regimen.

Item	load	Groups (group)	Times	Intermittent period
Squats	40 kg	3	6	60 s
pull-up	10 kg	3	6	60 s
single-leg deadlift Bosu ball	6 kg	3	8 repetitions per leg	60 s
Lift one arm while in a kneeling position	7.5 kg	3	8 repetitions per leg	60 s
Replicates an inverted water push-up	Elastic bands	3	15	60 s
Kneeling rotation	6 kg	3	8 repetitions per leg	60 s
Alternating leg lifts on the BOSU ball	1 kg	3	15 repetitions per leg	60 s
Prone back stretches	6 kg	3	15	60 s

### Inspiratory muscle function test

IM-related parameters, including the S-Index、peak inspiratory flow (PIF) and the maximum inspiratory pressure (MIP), were assessed using the PowerBreathe KH2 device. Initially, the supporting software Breathe Link was installed on a computer, and the pertinent information for all 14 subjects was entered into the software. Subsequently, the PowerBreathe KH2 was linked to the computer, and the software interface was configured for the testing process. The system was configured to initiate a test mode, which entailed 30 consecutive forceful inhalations without any time constraints. The software was designed to detect the shaded segment within the inhalation curve, displaying the maximum value from the previous test, providing a reliable means to quantify the subjects' basic data and assess the training effects. Throughout the test, participants were instructed to perform forceful inhalations while refraining from engaging in compensatory maneuvers.

### Resting pulmonary function test

Pulmonary function assessments were carried out using the CHEST H-101 resting pulmonary function analyzer, a Japanese-made device renowned for its capability to measure and analyze exhaled and inhaled gas flow and volume. This analysis provides crucial lung ventilation indices such as slow vital capacity (SVC), minute voluntary ventilation (MVV), and forced vital capacity (FVC), derived from the time-volume curve and flow-volume curve. The device is initiated to commence testing, and subsequently, sequential testing for SVC, FVC, and MVV is performed. Both SVC and FVC tests are conducted twice to ensure accurate results, while the MVV test is performed once. This standardized approach ensures consistency for comparative analysis.

### Maximal oxygen uptake and ventilation threshold testing

The study assessed the subjects' maximum oxygen uptake (VO2max) and ventilation threshold (VT) using a treadmill protocol. Prior to the test, participants were equipped with heart rate monitors, and safety belts, and underwent a 5 min warm-up on the treadmill. Upon donning the gas metabolism analyzer, the incremental exercise stress test is initiated.

The formal test began at a speed of 7 km/h, gradually increasing to 16 km/h, and then maintaining this speed until exhaustion. During the 7 km/h to 16 km/h phase, the speed increased by 1 km/h per minute, with the treadmill set at a 0% slope. Subsequently, the gradient increased by 1% per minute while maintaining the 16 km/h speed.

Test discontinuation criteria included reaching a respiratory quotient (RQ) at or near 1.15, achieving maximum heart rate (220-age) or a heart rate above 180 bpm, observing an oxygen uptake plateau, or exhibiting an inability to continue exercising. Real-time monitoring of metabolic and ventilation parameters was conducted using a gas metabolizer (smax58ce-sp, China), with data collected by a computer data analysis system. The VT was determined using the V-slope method, and the velocity at ventilatory thresholds (vVT) and the percentage of the ventilation threshold to the maximum oxygen uptake (VT/VO2max) were calculated. Additionally, the study evaluated the maximal oxygen uptake (VO2max), ventilatory threshold onset, and duration of escalating load exercise.

### Aquatic testing

In consideration of the energy supply characteristics and specific program demands in artistic swimming, we have selected the following test parameters to further validate its training effectiveness.

(1). 25 m torpedo: special swimming capacity;
(2). 50 m diving: breath-holding capacity.

### Statistical analysis

Following the verification of the experimental data, statistical analysis was conducted using SPSS 25.0 software. The normal distribution of continuous data was assessed with the Shapiro-Wilk test. Within-group differences were evaluated using the paired samples *t*-test, while differences between the two groups before and after the intervention were analyzed using the independent samples *t*-test. The presentation of experimental data included mean values expressed as mean ± standard deviation (Mean ± SD), and the calculation of 95% confidence intervals (95% CI) for each index. The significance level was set at *P* < 0.05 for statistical significance and *P* < 0.01 for extreme statistical significance.

## Results

No significant differences were observed between the CON and EXP groups with regard to age, height, and weight. Subjects in both groups exhibited no metabolic disorders, endocrine disorders, or abnormal pulmonary function. Additionally, before training, there were no significant disparities in respiratory muscle function, resting pulmonary function,maximal oxygen uptake and ventilation threshold,or specific exercise capacity test between the two groups.

### Inspiratory muscle function

Table 3 exhibits a notable increase in the inspiratory muscle strength index for both the CG and the TG groups post-stage training, yielding a *P*-value of <0.01. Furthermore, the peak inspiratory flow rate experienced a substantial increase solely within the TG group, with a *P*-value of <0.01, while no significant change was observed in the CG group. Upon comparing the groups, the inspiratory muscle strength index of the TG group displayed a significant increase post-stage training, with a *P*-value of <0.05.

**Table 3:** The impact of combining inspiratory muscle resistance with strength training on parameters related to inspiratory muscle strength.

Indexs	Groups	Pretest	Posttest
S-index (cmH2O)	CG (*n* = 7)	96.9 ± 4.5	115.4 ± 7.9**
	TG (*n* = 7)	100.9 ± 6.9	126.2 ± 7.6**^,#^
PIF (L/S)	CG (*n* = 7)	5.02 ± 0.50	6.36 ± 0.66
	TG (*n* = 7)	4.94 ± 0.36	6.72 ± 0.46**

Longitudinal comparison in the same group (compared with pretest results).

* With significant differences (*P* < 0.05).

** With very significant differences (*P* < 0.01); Difference between CON and EXP.

^#^ With significant differences (*P* < 0.05).

### Resting pulmonary function

As depicted in Table 4, both the CG and TG groups displayed a significant increase in spirometry and forced spirometry following the stage training, with a *P*-value of <0.01. Additionally, the TG group exhibited a significant increase in ventilation per minute, with a *P*-value of <0.05. Furthermore, the inter-group analysis revealed a significant increase in ventilation per minute in the TG group after the stage training, with a *P*-value of <0.05.

**Table 4:** The impact of combining inspiratory muscle resistance with strength training on parameters related to resting pulmonary function.

Group	SVC (L)		FVC (L)		MVV (L/min)	
	Pretest	Posttest	Pretest	Posttest	Pretest	Posttest
CG	4.27 ± 0.26	4.56 ± 0.27**	3.55 ± 0.26	3.93 ± 0.16**	110.03 ± 7.26	127.59 ± 7.57
TG	4.34 ± 0.31	4.78 ± 0.40**	3.71 ± 0.15	4.20 ± 0.23**	109.08 ± 4.54	133.11 ± 11.57*^,#^

Longitudinal comparison in the same group (compared with pretest results).

* With significant differences (*P* < 0.05).

** With very significant differences (*P* < 0.01); Difference between CON and EXP.

^#^ With significant differences (*P* < 0.05).

### Aerobic capacity

Based on the data in Table 5, it is observed that the CG group exhibited a significant increase only in tidal volume (*P* < 0.05). Conversely, the TG group showed significant increases in both the ventilation threshold and VO2 (*P* < 0.05), as well as a significant rise in tidal volume (*P* < 0.01). Furthermore, the corresponding running speed at which the VO2 maxima was reached demonstrated a significant increase in the inter-group comparison (*P* < 0.05).

**Table 5:** The impact of combining inspiratory muscle resistance with strength training on parameters related to aerobic capacity.

Index	Unit	CG (*n* = 7)		TG (*n* = 7)	
		Pretest	Posttest	Pretest	Posttest
VT	ml/min/kg	38.0 ± 4.8	39.8 ± 7.1	39.6 ± 4.7	42.7 ± 5.9
VT/VO2max (%)	%	77.9 ± 6.6	79.2 ± 6.6	77.3 ± 6.2	80.4 ± 5.1
vVT	km/h	12.0 ± 0.8	12.2 ± 0.8	12.0 ± 1.0	12.6 ± 0.8*
VO2max	ml/min/kg	48.7 ± 4.0	50.4 ± 6.3	51.2 ± 4.9	53.0 ± 5.0
vVO2max	km/h	15.5 ± 1.5	16.4 ± 1.1	16.3 ± 1.0	16.6 ± 1.1^#^
VEmax	L/min/kg	1.9 ± 0.2	1.9 ± 0.2	2.0 ± 0.3	2.0 ± 0.2
Vtex	ml/kg	31.8 ± 3.2	35.9 ± 4.9*	33.0 ± 4.1	37.7 ± 4.1**

Longitudinal comparison in the same group (compared with pretest results).

* With significant differences (*P* < 0.05).

** With very significant differences (*P* < 0.01); Difference between CON and EXP.

^#^ With significant differences (*P* < 0.05).

### Aquatic tests

As indicated in Table 6, both the CG and TG groups exhibited a significant improvement in the 50-meter diving and 25-meter torpedo scores after the stage training, with a *P*-value of <0.05. Additionally, a significant difference was observed in the 25-meter torpedo scores of the TG group when comparing between the groups, with a *P*-value of <0.05.

**Table 6:** The impact of combining inspiratory muscle resistance with strength training on parameters related to specialized tests.

Index	Group	Pretest	Posttest
50 m diving (s)	CG (*n* = 7)	35.2 ± 1.7	33.9 ± 2.5*
	TG (*n* = 7)	36.1 ± 0.9	33.7 ± 0.7**
25 m torpedo (s)	CG (*n* = 7)	23.5 ± 0.6	22.9 ± 0.5*
	TG (*n* = 7)	23.8 ± 0.7	22.0 ± 0.7**^,#^

Longitudinal comparison in the same group (compared with pretest results).

* With significant differences (*P* < 0.05).

** With very significant differences (*P* < 0.01); Difference between CON and EXP.

^#^ With significant differences (*P* < 0.05).

## Discussion

With the continuous development of the sport, artistic swimming, and the athletes' competitive level, require the choreography of artistic swimming to develop in the direction of faster, more difficult and more intense. The respiratory pattern of artistic swimming includes a rapid forced inspiratory phase and a prolonged breath-holding apnea phase while submerged. This entails a high metabolic demand and recurrent apnea in the aquatic environment leading to bradycardia and a suppressed hypoxic respiratory response (11). Consequently, this condition can stress the respiratory system through lung hyperinflation, hypoxemia, and mechanical loading (2). Following the implementation of updated regulations in artistic swimming, the demands for athletes' underwater breath-holding capacity have intensified.The breath-holding and immersion aspects in artistic swimming suggest a decrease in gas exchange and an increase in physiological stress during exercise (12–14).

The available research highlights that inspiratory muscle training is effective in enhancing gas exchange capacity and diminishing inspiratory muscle fatigue. This, in turn, improves lung function and aerobic capacity, ultimately resulting in enhanced exercise performance1–11.According to the studies (15), specific training following the principles of skeletal muscle training improves diaphragm strength, leading to enhanced inspiratory muscle strength through Inspiratory Muscle Resistance Training (IMRT). This follows the principles of stretch reflex and the length-tension relationship, resulting in reflexive strengthening of the expiratory muscles, accelerating the contraction rate of the entire breathing process, and improving lung ventilation efficiency, consistent with previous research (16–18). The improved lung ventilation efficiency enhances the body's oxygen-carrying capacity and increases oxygen uptake during exercise, consequently improving aerobic exercise capacity.

This study aims to explore the impact of a five-week inspiratory muscle resistance combined with strength training on the pulmonary function and exercise capacity of artistic swimmers.After different intensities of inspiratory muscle resistance combined with strength training, the lung function and inspiratory muscle strength of the subjects in both groups were significantly improved, which was reflected in the significant increase in inspiratory muscle strength index, vital capacity and forced vital capacity after the stage training. The peak inspiratory flow rate and minute ventilation in the TG group were significantly improved after training. The comparison between groups showed that the inspiratory muscle strength index and ventilation per minute were significantly improved, suggesting that the combination of high-intensity inspiratory muscle resistance training and strength training had a better effect on improving the strength of inspiratory muscles and the endurance level of respiratory muscles in artistic swimmers. The increase in inspiratory muscle strength results in greater force production during diaphragm and other inspiratory muscle contractions, leading to increased diaphragm displacement during breathing, consequently expanding chest volume and promoting lung expansion (14, 15, 19, 20). Enhanced inspiratory muscle strength enables quicker muscle contraction and greater force production, boosting the explosive power of expiratory muscles, increasing tidal volume per inhalation, and the corresponding lung ventilation, all while maintaining a consistent respiratory rate (14, 15, 19, 21). Moreover, improved strength and endurance of the respiratory muscles enable more effective maintenance of a stable respiratory rate and depth, ultimately enhancing lung ventilation efficiency.

And our study found that the aerobic exercise capacity of athletes in the TG group was significantly improved after the stage of inspiratory muscle resistance combined with strength training, and the improvement effect was better than that of the CG group, which was reflected in the fact that after the stage training, the tidal volume of the CG group and the TG group was significantly improved, and the corresponding running speed of the TG group when the ventilation threshold and maximum oxygen uptake were significantly increased. In addition, comparisons between groups showed that the TG group had a significant increase in running speed when the VO2 maxima was reached.The TG group had a significantly higher increase in running speed when reaching VO2 maxima than the CG group, and it was believed that the form of inspiratory muscle resistance combined with strength training improved the efficiency of lung ventilation and the functional ability of respiratory muscles. In addition, the reduction of metabolites also reduces the stimulation of metabolic receptors of respiratory muscles and increases the threshold of metabolic reflex activation (15), thereby effectively improving the body's aerobic exercise capacity.

The primary aim of inspiratory muscle training is to establish a foundation for improving performance in aquatic specialized exercises (14, 15, 19–21). With this goal in mind, we have selected specific performance indicators for artistic swimming, compared the effects of two types of inspiratory muscle training in enhancing the specialized performance ability in artistic, and analyzed the correlation between the relevant indicators and the specialized performance metrics.

The analysis of the test results of 25-meter torpedo and 50-meter diving after stage training found that the special swimming ability of athletes was significantly improved after stage inspiratory muscle resistance and combined strength training, which was reflected in the fact that the 25-meter torpedo and 50-meter diving in the CG group and TG group were significantly improved after stage training. In addition, the comparison between groups showed that the 25-meter torpedo in the TG group was significantly improved, and it was believed that the stage of inspiratory muscle resistance combined with strength training significantly improved the breath-holding ability and special swimming ability of artistic swimmer.

Support for these findings can also be found in the work reported similar results on the positive impact of inspiratory muscle training on the specialized exercise ability of artistic swimmers. Additionally, Volianitis S's study (21) demonstrated that inspiratory muscle training (IMT) improved the performance in the 6 min exhaustion test and the 5,000-m test in rowers, reducing inspiratory muscle fatigue and dyspnea.

In summary, the observed enhancements in athletes' specialized exercise ability as a result of inspiratory muscle resistance training are linked to improved inspiratory muscle function and ventilation efficiency. Furthermore, the improvement in respiratory regulation ability, including breathing control and depth, is considered to be a contributing factor to these outcomes.

## Limitation

This study presents novel insights into the potential of inspiratory muscle training to enhance athletes' specific exercise abilities. However, it is important to note that the findings may be constrained by the small sample size. Despite meeting the requirements calculated by G-Power, the current sample size is small. Therefore, we are contemplating expanding the sample size in future studies to bolster the stability and reliability of the results.

## Conclusion

A five-week combined inspiratory muscle resistance and strength training regimen can significantly enhance the aerobic capacity of artistic swimmers, thereby establishing a basis for elevating the competitive standard in artistic swimming. Both high-intensity and low-intensity inspiratory muscle resistance combined with strength training can effectively enhance the inspiratory muscle function and overall cardiopulmonary capacity of athletes. Furthermore, high-intensity inspiratory muscle resistance combined with strength training demonstrates superior efficacy in enhancing the ability of artistic swimmers to optimize oxygen utilization within exercising muscles.

## Data Availability

The original contributions presented in the study are included in the article/Supplementary Material, further inquiries can be directed to the corresponding author.
